# Vision loss and visual hallucinations: the Charles Bonnet syndrome

**Published:** 2009-03

**Authors:** Paddy Ricard

**Affiliations:** Consultant editor for the *Revue de Santé Oculaire Communautaire*, International Centre for Eye Health, London School of Hygiene and Tropical Medicine, Keppel Street, London WC1N 7HT, UK.

**Figure:**
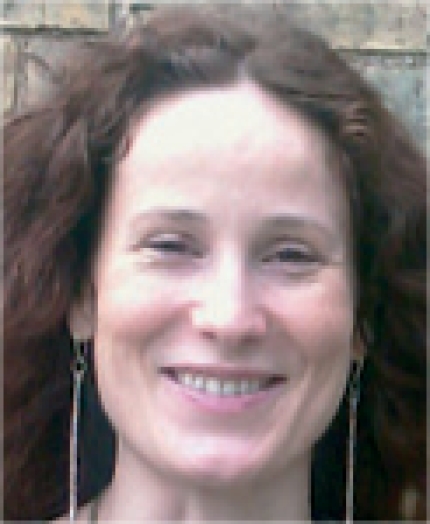


Around half the people presenting with acquired severe sight loss will at some point experience visual hallucinations. This is a little-known fact, not only amongst patients, but also amongst a significant number of health and eye care professionals. The condition is known as Charles Bonnet syndrome (CBS) and is named after the naturalist who first described this phenomenon in 1759.

Although visual hallucinations can be unsettling, current ignorance and lack of information about the condition lead to even greater distress in patients. They may believe that they are suffering from a mental condition, such as dementia, and that they are losing not only their sight, but also their mind.

The Royal College of Ophthalmologists^1^ and the Macular Disease Society (MDS)^2^ have now initiated a campaign to increase awareness of CBS amongst eye care staff. The many case studies collected by the MDS, such as the following, all tell a similar story.

## Case study

Mrs O had her first hallucination three years after been diagnosed with age-related macular degeneration (AMD), at the age of 82: she woke up in the middle of the night to find that her bedroom walls seemed covered in white fleece. She thought she was losing her mind and kept it to herself, though she was very distressed. When the hallucinations worsened, she decided to talk to medical staff. However, her general practitioner did not take her seriously, and the Accident & Emergency department of her local hospital referred her to a psychiatrist, suspecting that she suffered from dementia. Mrs O only found out about CBS after six months, through the MDS, and she immediately experienced a great relief. Since then, although she still finds some hallucinations frightening, she has found it much easier to cope with them.

**Figure:**
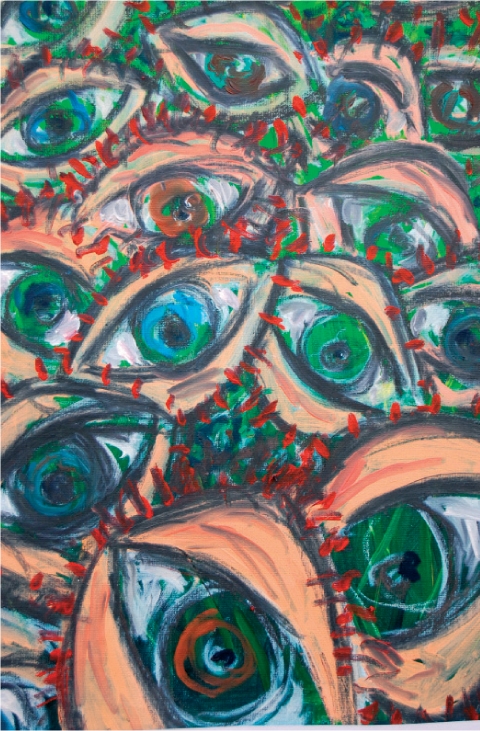
Artist Cecil Riley, who suffers from CBS, depicts his visions in his paintings. UK

Cecil Riley

**Figure:**
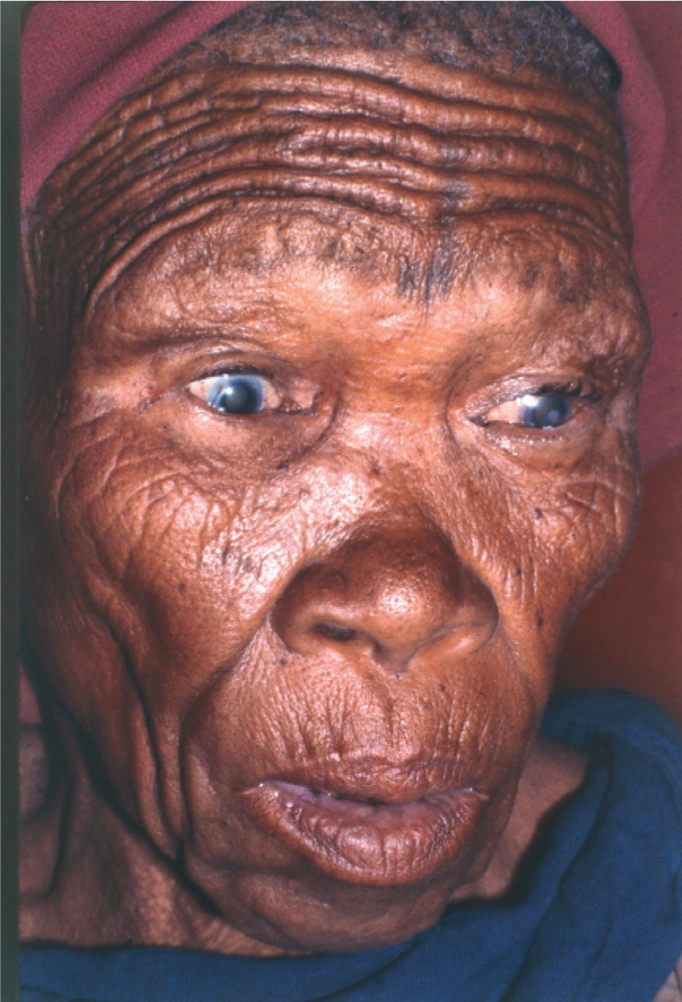
Around half the people suffering from severe visual loss will experience visual hallucinations. BOTSWANA

Nick Astbury

## Features of CBS

CBS occurs mostly in people who have developed severe visual loss in **both** eyes involving **central vision.** This situation is especially likely to occur in developing countries, where people may wait for sight loss before consulting and where advanced bilateral cataract is common. Causes of severe visual loss include:

- cataract
- glaucoma
- trauma
- macular degeneration
- diabetic retinopathy with maculopathy
- retinal detachment.

Here are some of the features of CBS:

- Patients experience complex visual hallucinations, i.e. fully formed images.
- Hallucinations include: patterns (brickwork, grids, etc.), letters, people (sometimes distorted or incomplete), animals, objects, and landscapes.
- There is no sound associated with these hallucinations.
- These hallucinations are due to impulses from the visual cortex in the absence of visual stimulation.
- Hallucinations may start occurring soon after the onset of visual loss, but they can sometimes appear up to ten years later.

## Information and reassurance

There is currently no treatment for CBS. However, health care personnel can still play a crucial role in alleviating anxiety experienced by patients, by informing them about the condition and reassuring them on their mental state. The eye care team may offer the following reassuring statements^3^^,^^4^:

- It is estimated that 50-60% of people suffering from severe visual loss will experience visual hallucinations.
- These visual hallucinations seem to abate after a while, usually after 18 months for 60% of patients.
- The visual hallucinations are a purely visual symptom and are not due to any mental health problem.
- Although there is no treatment, some patients find ways of controlling their hallucinations or of distinguishing between a real sight and a hallucination.

Tricks' reported by sufferers include: going into a brighter environment, creating a distraction, looking directly at the images, some form of eye movement, etc. (not all of these suggestions may work for all patients).

## Conclusion

It is particularly important that all staff know about CBS, including receptionists, so that they do not turn patients away needlessly or doubt the veracity of their statements (or indeed their sanity).

A recent survey showed that, amongst those suffering from CBS: 60% feared being labelled as insane if they admitted to hallucinations, only 30% had ever revealed their condition to anyone else, and 30% lived in fear of impending insanity.^3^

CBS is still largely under-recognised, and further awareness of the condition can only encourage patients to report their fears. It is also important to forewarn patients that such hallucinations may occur.

Once informed, patients will not be so anxious about their mental health. They may develop ways of managing their hallucinations and may become more confident in using their residual vision.
